# The age again in the eye of the COVID-19 storm: evidence-based decision making

**DOI:** 10.1186/s12979-021-00237-w

**Published:** 2021-05-20

**Authors:** María C. Martín, Aurora Jurado, Cristina Abad-Molina, Antonio Orduña, Oscar Yarce, Ana M. Navas, Vanesa Cunill, Danilo Escobar, Francisco Boix, Sergio Burillo-Sanz, María C. Vegas-Sánchez, Yesenia Jiménez-de las Pozas, Josefa Melero, Marta Aguilar, Oana Irina Sobieschi, Marcos López-Hoyos, Gonzalo Ocejo-Vinyals, David San Segundo, Delia Almeida, Silvia Medina, Luis Fernández, Esther Vergara, Bibiana Quirant, Eva Martínez-Cáceres, Marc Boiges, Marta Alonso, Laura Esparcia-Pinedo, Celia López-Sanz, Javier Muñoz-Vico, Serafín López-Palmero, Antonio Trujillo, Paula Álvarez, Álvaro Prada, David Monzón, Jesús Ontañón, Francisco M. Marco, Sergio Mora, Ricardo Rojo, Gema González-Martínez, María T. Martínez-Saavedra, Juana Gil-Herrera, Sergi Cantenys-Molina, Manuel Hernández, Janire Perurena-Prieto, Beatriz Rodríguez-Bayona, Alba Martínez, Esther Ocaña, Juan Molina

**Affiliations:** 1 Centro de Hemoterapia y Hemodonación de Castilla y León, Valladolid, Spain; 2 Department of Immunology and Allergology, Hospital Universitario Reina Sofía-Instituto de Investigación Biomédica de Córdoba (IMIBIC), Avd. Menéndez Pidal s/n, 14004 Córdoba, Spain; 3 Department of Microbiology and Immunology, Hospital Clínico Universitario, Valladolid, Spain; 4 Department of Immunology, Hospital Universitario Son Espases-Human Immunopathology Research Laboratory, Institut d’Investigació Sanitària de les Illes Balears (IdISBa), Palma de Mallorca, Spain; 5 Department of Immunology, Hospital Clínico Universitario, Salamanca, Spain; 6 Department of Immunology, Fundación Jiménez Díaz, Madrid, Spain; 7 Department of Immunology, Hospital Universitario de Badajoz, Badajoz, Spain; 8 Department of Immunology, Hospital Universitario Marqués de Valdecilla, Santander, Spain; 9 Laboratory of Immunology, Complejo Hospitalario Nuestra Señora de la Candelaria, Santa Cruz de Tenerife, Spain; 10 Laboratoy of Immunology and Genetics, Hospital San Pedro de Alcántara, Cáceres, Spain; 11 Department of Immunology, Hospital Germans Trias i Pujols, Barcelona, Spain; 12 Department of Immunology, Hospital de Cruces, Baracaldo, Spain; 13 Department of Immunology, Hospital Universitario La Princesa, Madrid, Spain; 14 Department of Immunology, Hospital Torrecárdenas, Almería, Spain; 15 Department of Immunology, Hospital de Donostia, San Sebastián, Spain; 16 Unit of Immunology, Hospital General Universitario, Albacete, Spain; 17 Laboratory Unit, Hospital General, Alicante, Spain; 18 Department of Immunology, Complejo Hospitalario, La Coruña, Spain; 19 Unit of Immunology, Hospital Universitario Insular-Materno Infantil, Las Palmas de Gran Canaria, Spain; 20 Department of Immunology, Hospital General Universitario e Instituto de Investigación Sanitaria, “Gregorio Marañón”, Madrid, Spain; 21 Department of Immunology, Hospital Universitario Vall d’Hebron, Barcelona, Spain; 22 Laboratory Unit, Hospital Juan Ramón Jiménez, Huelva, Spain; 23 Laboratory Unit, Complejo Hospitalario, Jaén, Spain

**Keywords:** Severe acute respiratory syndrome coronavirus 2, COVID-19, Immunosenescence, Lockdown, Immunity, Renin-angiotensin-aldosterone system inhibitors, Cut-off points, Lymphocytes, Area under the curve

## Abstract

**Background:**

One hundred fifty million contagions, more than 3 million deaths and little more than 1 year of COVID-19 have changed our lives and our health management systems forever. Ageing is known to be one of the significant determinants for COVID-19 severity. Two main reasons underlie this: immunosenescence and age correlation with main COVID-19 comorbidities such as hypertension or dyslipidaemia. This study has two aims. The first is to obtain cut-off points for laboratory parameters that can help us in clinical decision-making. The second one is to analyse the effect of pandemic lockdown on epidemiological, clinical, and laboratory parameters concerning the severity of the COVID-19. For these purposes, 257 of SARSCoV2 inpatients during pandemic confinement were included in this study. Moreover, 584 case records from a previously analysed series, were compared with the present study data.

**Results:**

Concerning the characteristics of lockdown series, mild cases accounted for 14.4, 54.1% were moderate and 31.5%, severe. There were 32.5% of home contagions, 26.3% community transmissions, 22.5% nursing home contagions, and 8.8% corresponding to frontline worker contagions regarding epidemiological features. Age > 60 and male sex are hereby confirmed as severity determinants. Equally, higher severity was significantly associated with higher IL6, CRP, ferritin, LDH, and leukocyte counts, and a lower percentage of lymphocyte, CD4 and CD8 count. Comparing this cohort with a previous 584-cases series, mild cases were less than those analysed in the first moment of the pandemic and dyslipidaemia became more frequent than before. IL-6, CRP and LDH values above 69 pg/mL, 97 mg/L and 328 U/L respectively, as well as a CD4 T-cell count below 535 cells/μL, were the best cut-offs predicting severity since these parameters offered reliable areas under the curve.

**Conclusion:**

Age and sex together with selected laboratory parameters on admission can help us predict COVID-19 severity and, therefore, make clinical and resource management decisions. Demographic features associated with lockdown might affect the homogeneity of the data and the robustness of the results.

**Supplementary Information:**

The online version contains supplementary material available at 10.1186/s12979-021-00237-w.

## Background

SARS-CoV-2 infection became widespread [1], being possibly the worst trouble worldwide, as its effects have altered virtually any feature in our lives. Health, economy and individual freedom are seriously threatened all around the world. Almost 16 months after the first diagnosed case, several waves and strains have hit global health. The virus has infected 152.974.685 people and killed 3.204.478 with an overall case-fatality rate of 2.09% [2]. Identifying risk or severity factors for COVID-19 will help clinicians and clinical managers to make decisions about the best therapy [3], and the kind and amount of resources necessary to face new waves [4]. Severity factors might be related to health-based restrictions and should be considered before making public health decisions [5, 6].

Since the early days of the pandemic, enormous efforts have been made to identify epidemiological and clinical factors that can predict the severity of the disease [7–15]. These efforts have firmly established age as one of the crucial elements along with comorbidities closely associated with age, most notably hypertension, diabetes, and obesity [16–23]. Independently, the male sex has also been described to be related to a severe evolution [16, 18–20, 22, 23]. Likewise, the analytical parameters related to an exacerbated inflammatory state and an exhausted adaptive immune system have been described in association with the most severe forms [16, 24–26]. Despite the similarity in the overall description of the parameters associated with the severity of COVID-19 disease, there are notable differences in the risk factors and the analytical parameter values, among the articles published [27]. The causes for these differences can rely on the geographical origin of the study populations, the different study designs and the severity criteria adopted. Additionally, some elements, which are not usually reflected in the scientific literature, might influence the pandemic landscape. One of these elements would be the effect of the pandemic lockdown restrictions, with the meaning of “stay-at-home orders” in the patient baseline characteristics. These differences may be especially relevant in defining cut-off points for analytical parameters that could be extrapolated to different populations and different pandemic moments. The objective of this study is twofold. First, to obtain cut-off points for laboratory parameters that can help us in clinical decision-making. Secondly, to evaluate the effect that confinement may have on the patient demographic and clinical characteristics. This study aims to analyse the effect of pandemic lockdown on epidemiological, clinical, and laboratory parameters concerning the severity of the COVID-19; for this purpose, we have compared the baseline demographic and clinical characteristics as related to the severity of COVID-19 inpatients infected before complete lockdown, with those of patients admitted to hospital during close lockdown period.

## Results

A total of 257 inpatients from 13 Spanish Hospitals with SARS-CoV-2 infection were included. Mild cases accounted for 14.4, 54.1% moderate and 31.5% severe (Table 1), with 32.5% of home contagions, 26.3% community transmissions, 22.5% nursing home contagions and 8.8% corresponding to frontline workers.

**Table 1:** Baseline characteristics of the study population

Clinical and demographic characteristics	All patients *n* = 257; (%)					
Severity						
Mild	37 (14.4)					
Moderate	139 (54.1)					
Severe	81 (31.5)					
Sex						
Male	148 (57.6)					
Female	109 (42.4)					
Hypertension	121 (49.2)					
ACEI^a^ intake						
No	74 (67.9)					
Yes	35 (32.1)					
ARB^a^ intake						
No	74 (67)					
Yes	36 (33)					
Dyslipidaemia	102 (41.6)					
Statins intake						
No	57 (52.8)					
Yes	51 (47.2)					
Diabetes	68 (27.6)					
Obesity	34 (16.9)					
Primary Immunodeficiency	2 (0.9)					
Secondary Immunodeficiency	41 (18.1)					
Epidemiological background						
Nursing home resident	36 (22.5)					
Live-in relative	52 (35.5)					
Frontline worker	14 (8.8)					
Community transmission	42 (26.3)					
	Ref.v^b^	n	Mean	Median	SD^c^	IQR^d^
Age		257	66.0	68.0	17.0	54–90
**Laboratory data on admission**						
IL6^e^ (pg/mL)	< 4.4	139	62.2	29.9	94.4	10–296.6
CRP^f^ (mg/L)	< 10	242	105	47	246	10.8–324.6
Ferritin (ng/mL)	20–250	197	882	499	111	211–2718
D-dimer (ng/mL)	< 500	237	2532	741	10,683	410–6258
LDH^g^ (U/L)	120–246	234	336	289	168	212–681
Days from onset to admission		240	8	7	5	4–16
Leucocyte count (cells*10^3^/μL)	4–12.4	252	8.31	6.60	7.99	4.90–17.60
Neutrophil count (cells*10^3^/μL)	1.9–8	252	6.20	5.06	4.14	3.26–15.07
Lymphocyte count (cells*10^3^/μL)	0.9–5	252	2.18	1.00	10.75	0,7-2,59
Lymphocyte %	19–48	232	17.30	14.50	12.00	9.5–40.3
CD3 + CD4+ %	25–65	76	44.20	45.80	13.20	36.6–67.9
CD3 + CD4+ count (cells*10^6^/μL)	500–1400	75	541	460	365	257–1263
CD3 + CD8+ %	12–40	76	21.70	21.10	10.50	14.5–40
CD4 + CD8+ count (cells*10^6^/μL)	250–1000	75	268	182	234	117–685
CD19+ %	5–20	68	14.20	13.30	8.30	8.1–32.8
CD19+ count (cells*10^6^/μL)	100–500	67	164	127	151	66–409
Natural Killer %	5–20	68	16.30	14.40	9.00	9–36.8
Natural Killer count (cells*10^6^/μL)	50–500	64	171	148	107	103–335
Immunoglobulin G (mg/dL)	650–1600	60	961.0	924.0	431.0	728–1629
Immunoglobulin A (mg/dL)	40–350	60	264.0	228.0	152.0	162–587
Immunoglobulin M (mg/dL)	50–300	60	105.0	89.0	83.0	70–256
C3 (mg/dL)		71	133.0	127.0	46.0	108–225
C4 (mg/dL)		70	30.0	28.0	13.0	23–56
Total days in hospital		245	16	12	11	7–38

*Abbreviations*: *RASB*^a^ Renin-angiotensin system blockers, *Ref.v*^b^ Reference values, *SD*^c^ Standard deviation, *IQR*^d^ Interquartile range, *IL6*^e^ Interleukin 6, *CRP*^f^ C-reactive protein, *LDH*^g^ Lactate dehydrogenase

Descriptive baseline characteristics of the population (valid n, frequencies, percentages, mean, median, standard deviation and interquartile range) are shown in Table 1. Categorical variables stratified by severity are shown in Table 2.

**Table 2:** Risk factors by severity n (%)

	Mild	Moderate	Severe
	n (%)	n (%)	n (%)
**Age (*p*** **= 0.046)**			
< 30	0(0)	7(87.5)	1(12.5)
30–45	3(13)	16(69.6)	4(17.4)
45–60	12(19.7)	36(59)	13(21.3)
60–75	9(10.1)	45(50.6)	35(39.3)
> 75	13(17.1)	35(46.1)	28(36.8)
**Gender (*p*** **= 0.049)**			
Male	21(14.2)	73(49.3)	54(36.5)
Female	16(14.7)	66(60.6)	27(24.8)
**Hypertension**			
No	15(12)	74(59.2)	36(28.8)
Yes	19(15.7)	59(48.8)	43(35.5)
**ACEI**^**a**^ **intake**			
No	6(8)	42(56.8)	26(35.1)
Yes	7(20)	15(42.9)	13(37.1)
**ARB**^**b**^ **intake**			
No	8(11)	37(50.7)	28(38.4)
Yes	4(11.1)	19(52.8)	13(36.1)
**Dyslipidaemia**			
No	19(13.3)	84(58.7)	40(28)
Yes	13(12.7)	49(48)	40(39.2)
**Statins intake**			
No	19(12.8)	88(59.1)	42(28.2)
Yes	9(11.3)	39(48.8)	32(40)
**Diabetes**			
No	23(12.9)	102(57.3)	53(29.8)
Yes	9(13.2)	33(48.5)	26(38.2)
**Obesity**			
No	20(12)	95(56.9)	52(31.1)
Yes	3(8.8)	17(50)	14(41.2)
**Primary Immunodeficiency**			
No	27(11.6)	130(55.8)	76(32.6)
Yes	0(0)	2(100)	0(0)
**Secondary Immunodeficiency**			
No	23(12.4)	103(55.7)	59(31.9)
Yes	3(7.3)	24(58.5)	14(34.1)
**Epidemiological background (*p*** **= 0.001)**			
Nursing home resident	2(5.6)	19(52.8)	15(41.7)
Live-in relative	10(19.2)	37(71.2)	5(9.6)
Frontline worker	0(0)	11(78.6)	3(21.4)
Community transmission	6(14.3)	16(38.1)	20(47.6)

*Abbreviations*: *ACEI*^a^ Angiotensin-converting enzyme inhibitors, *ARB*^b^ Angiotensin II receptor blockers

Males accounted for 58% of cases. Ages in our cohort ranged from 18 to 97 years, with a median of 68 years (IQR 54–90). Concerning comorbidities, 16.9% had obesity, 15.8% were smoker or ex-smokers, 49.2% had hypertension, 32.1% of them were treated with ACEIs (angiotensin converting enzyme inhibitors) and 33% with ARBs (angiotensin receptor blockers). 41.6% had dyslipidaemia and 27.6% suffered diabetes mellitus. The presence of immunodeficiency was most often secondary to other processes, such as a transplantation or chemotherapy treatment; it accounted for 19% cases (*n* = 45) as seen in Table 1.

Age above 60 (*p* = 0.046), male gender (*p* = 0.049) and institution or community transmission (*p* < 0.001) arose as severity determinants in our series (Table 2).

Neither hypertension nor the use of renin-angiotensin system blockers (RAABs) was significantly associated with severity. Mild cases accounted for 10.1% of patients with age ranged between 60 and 75 years, and 17.1% of patients over 75, whereas only 5.6% of home nursing cases were mild.

**Fig. 1:**
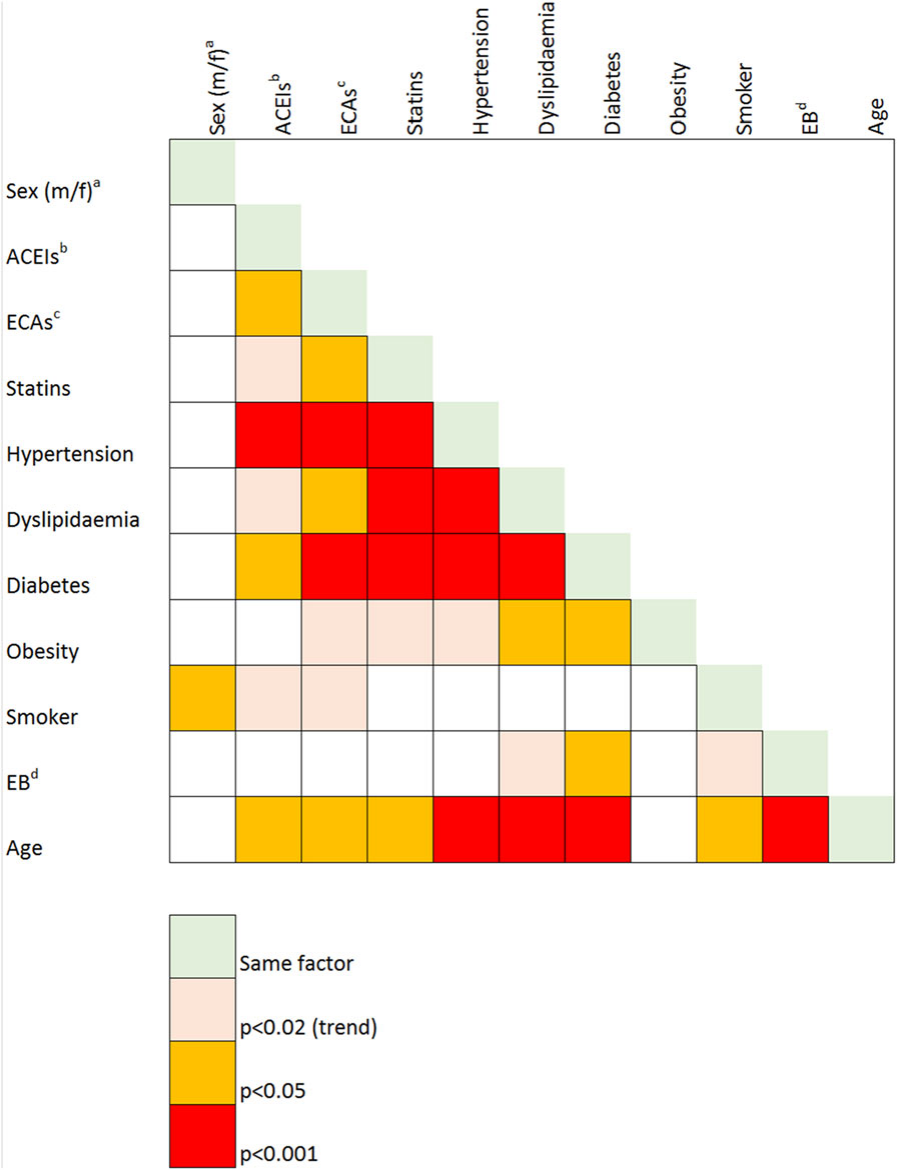
Severity factors and comorbidities interactions. Legend. Pearson’s Chi Squared *p*-values. Abbreviations: Sex(m/f)^a^: Sex (male/female); ACEIs^b^: angiotensin conversor enzyme inhibitors; ARBs^c^: angiotensin II receptor blockers; EB^d^: epidemiological background

Most comorbidities were age-related, such as hypertension, dyslipidaemia, diabetes and primary immunodeficiency. Smoking status was both age and sex-related (Table 3) (Fig. 1).

**Table 3:** Influence of age and gender on comorbidities

	Age					Gender	
	< 30	30–45	45–60	60–75	> 75	Male	Female
	n (%)	n (%)	n (%)	n (%)	n (%)	n (%)	n (%)
**Hypertension**^**a**^							
No	7(5.6)	19(15.2)	41(32.8)	41(32.8)	17(13.6)	67(53.6)	58(46.4)
Yes	0(0)	2(1.7)	17(14)	45(37.2)	57(47.1)	73(60.3)	48(39.7)
**Dyslipidaemia**^**a**^							
No	7(4.9)	17(11.9)	45(31.5)	47(32.9)	27(18.9)	81(56.6)	62(43.4)
Yes	0(0)	4(3.9)	15(14.7)	38(37.3)	45(44.1)	59(57.8)	43(42.2)
**Obesity**							
No	5(3)	14(8.4)	42(25.1)	56(33.5)	50(29.9)	103(61.7)	64(38.3)
Yes	1(2.9)	2(5.9)	7(20.6)	18(52.9)	6(17.6)	17(50)	17(50)
**Diabetes**^**a**^							
No	7(3.9)	18(10.1)	55(30.9)	57(32)	41(23)	102(57.3)	76(42.7)
Yes	0(0)	3(4.4)	5(7.4)	31(45.6)	29(42.6)	40(58.8)	28(41.2)
**Smoker**^**a,b**^							
No	4(2.2)	17(9.1)	50(26.9)	65(34.9)	50(26.9)	101(54.3)	85(45.7)
Yes	3(12)	1(4)	7(28)	6(24)	8(32)	20(80)	5(20)
Ex	0(0)	0(0)	0(0)	8(80)	2(20)	8(80)	2(20)
**Primary immunodeficiency**^**a**^							
No	7(3)	21(9)	59(25.3)	83(35.6)	63(27)	133(57.1)	100(42.9)
Yes	1(50)	0(0)	0(0)	0(0)	1(50)	1(50)	1(50)
**Secundary immunodeficiency**							
No	5(2.7)	16(8.6)	52(28.1)	60(32.4)	52(28.1)	98(53)	87(47)
Yes	2(4.9)	3(7.3)	6(14.6)	21(51.2)	9(22)	28(68.3)	13(31.7)
**Epidemiological background**							
NHR	1(2.8)	3(8.3)	6(16.7)	8(22.2)	18(50)	18(50)	18(50)
LIR	1(1.9)	5(9.6)	15(28.8)	16(30.8)	15(28.8)	31(59.6)	21(40.4)
FW	1(7.1)	1(7.1)	7(50)	4(28.6)	1(7.1)	5(35.7)	9(64.3)
CT	3(6.7)	7(15.6)	12(26.7)	23(51.1)	13(51.1)	38(65.5)	20(34.5)

All *p*-values either age^a^ or gender^b^ were < 0.001

*Abbreviations*: *NHR* Nursing home resident, *LIR* Live-in relative, *FW* Frontline worker, *CT* Community transmission

On admission, the median of laboratory parameters, IL6, CRP, ferritin, D-dimer and lactate dehydrogenase (LDH) were above reference ranges; but both, percentage and median lymphocyte counts were under reference ranges (Table 1). Higher severity was significantly associated with higher IL6, CRP, ferritin, LDH, and leukocyte counts, and lower percentage and lymphocyte counts (Table 4). Results from 76 cases with data of lymphocyte subpopulations on admission showed that higher severity was significantly associated with lower CD4 and CD8 counts (Table 4).

**Table 4:** Age and Laboratory results by COVID-19 severity

	Severity *p*-value	n	Mean	Median	SD^a^	IQR^b^
**Age**	0.002					
Mild		37	68.22	68	16.64	54–82
Moderate		138	62.87	66	16.84	49.32–76
Severe		80	70.59	70	13.19	61.5–80.5
***On admission***						
**IL6**^**c**^ **(pg/mL)**	< 0.001					
Mild		11	25.01	10.6	32.31	5.2–37.1
Moderate		88	44.85	29.46	56.46	8.41–56.5
Severe		40	110.47	43.45	143.75	20.05–143.62
**CRP**^**d**^ **(mg/L)**	0.006					
Mild		34	43.87	22.35	54.88	8.16–58.4
Moderate		135	80.74	37.8	200.46	6.8–103.75
Severe		73	177.13	100.6	344.21	43.3–195.3
**Ferritin (ng/mL)**	0.002					
Mild		22	474.00	256.8	540.30	197–535
Moderate		120	731.87	459.75	907.18	181–925
Severe		55	1373.10	929	1488.30	366.4–1805
**D-dimer (ng/mL)**						
Mild		30	1234.90	684	2069.30	373–1297
Moderate		134	1652.22	715.5	5786.79	431–1150
Severe		73	4680.19	800	17,425.42	462–1990
**LDH**^**e**^ **(U/L)**	< 0.001					
Mild		31	215.39	206	62.63	172–243
Moderate		128	328.92	287	160.07	213.5–408.5
Severe		75	396.96	354	183.43	263–507
Leucocyte count (cells*10^**3**^**/μL)**	0.011					
Mild		34	424.62	6.39	1813.47	5.53–8.78
Moderate		137	190.92	6.37	1098.34	4.6–8.75
Severe		81	280.85	8.25	1441.86	5.5–12.98
Neutrophil count (cells*10^**3**^**/μL)**						
Mild		34	286.69	4.85	1244.32	3–6.85
Moderate		137	129.54	4.8	782.33	3.17–7
Severe		81	220.16	7	1147.11	4.19–11.58
Lymphocyte count (cells*10^**3**^**/μL)**	0.005					
Mild		34	1.44	1.29	0.78	0,84-1,78
Moderate		137	2.1	1.06	9.07	0,7-1,39
Severe		81	1	0.91	0.59	0,68-1,15
**Lymphocyte %**	0.005					
Mild		34	21.92	18.85	12.74	14–28.2
Moderate		119	18.82	15	12.64	10.9–25
Severe		79	13.10	11	9.27	6.6–16.7
**CD3 + CD4+ %**						
Mild		9	51.17	51	11.79	43.1–56.7
Moderate		48	43.88	45.76	12.71	37.43–51.525
Severe		19	41.78	41	14.46	34.32–52.74
CD3 + CD4+ count (cells*10^**3**^**/μL)**	0.007					
Mild		9	729.87	565	445.12	372.015–1035
Moderate		48	586.66	516	365.49	289.252–818.5
Severe		18	325.35	293	210.64	185.934–466.519
**CD3 + CD8+ %**						
Mild		9	20.45	24.4	10.09	14.79–27.1
Moderate		48	21.95	21.84	9.48	14.83–27.05
Severe		19	21.71	18	13.23	12.34–28.9
CD4 + CD8+ count (cells*10^**3**^**/μL)**	0.018					
Mild		9	263.29	177	205.19	143.616–269.955
Moderate		48	283.92	214	195.35	132.5–412.5
Severe		18	226.03	129.22	331.47	82–228
**CD19+ %**						
Mild		9	12.08	12.76	5.00	9.8–15.8
Moderate		44	13.98	13.47	7.91	7.4–17.18
Severe		15	16.16	14.5	10.81	9–20.68
CD19+ count (cells*10^**3**^**/μL)**						
Mild		9	166.59	145	108.67	76.23–241.74
Moderate		44	177.19	130	168.64	66.5–217.966
Severe		14	120.52	114.85	108.90	66–132.936
**Natural Killer %**						
Mild		9	12.91	11	6.85	7.7–15
Moderate		44	16.85	14.63	9.24	9.205–20.75
Severe		15	16.93	17.06	9.66	8.36–23.8
Natural Killer count (cells*10^**3**^**/μL)**						
Mild		9	159.68	132	89.67	103–169
Moderate		41	188.59	157	116.15	119–223
Severe		14	126.01	127.9	75.10	66–188
**IgG (mg/dL)**						
Mild		9	853.84	918	212.83	782–980
Moderate		31	981.27	950.3	296.30	772.14–1190
Severe		20	976.96	821	642.17	618.255–1048.5
**IgA (mg/dL)**						
Mild		9	284.40	239	206.15	175–277
Moderate		31	278.63	251	133.13	184.85–358
Severe		20	232.88	189.97	154.48	152.5–311
**IgM (mg/dL)**	0.009					
Mild		9	164.57	94	175.42	86.3–133
Moderate		31	103.63	98.7	53.67	71.59–132
Severe		20	81.39	84	43.07	46.45–112.95
**C3 (mgdL)**						
Mild		8	142.80	139	25.72	125–151.68
Moderate		38	135.03	127	50.55	108–152
Severe		25	126.91	125	44.84	90–153.28
**C4 (mgdL)**						
Mild		8	31.58	29.5	5.26	28.4–33.85
Moderate		37	30.52	29.3	11.72	23–39
Severe		25	28.30	25.7	15.40	23–29.8

*Abbreviations*: *SD*^a^ Standard deviation, *IQR*^b^ Interquartile range, *IL6*^c^ Interleukin 6, *CRP*^d^ C-reactive protein, *LDH*^e^ Lactate dehydrogenase

### Comparison of the two series: patients recruited on the very first days of pandemic vs. close confinement

Our group has previously published data on the risk factors and laboratory parameters of a multicentre series of patients admitted by COVID-19 during the first weeks of the pandemic [16]. A comparison of data corresponding to the close confinement (phase 2 from now on) with the previous series (phase 1 from now on) was performed.

Even with the same inclusion criteria, along with current data and the previous compilation, there were significantly less mild inpatients in phase 2 in comparison with phase 1 (*p* < 0.001). Age was significantly higher (*p* = 0.027) within the second period. More cases were reported to have dyslipidaemia (*p* < 0.001), a history of secondary immunodeficiency (*p* < 0.001) and fewer patients were on treatment with angiotensin II receptor blockers (*p* = 0.002) (Supplementary Tables 1 and 2).

Laboratory parameters such as IL-6, CRP and ferritin, although increased, were significantly lower during confinement than in the early months of the pandemic (*p* = 0.028, < 0.001 and < 0.001 respectively). In contrast, lymphopenia and declining CD8+ cell counts were more evident in the second phase but did not reach significance (Supplementary Table 2).

**Fig. 2:**
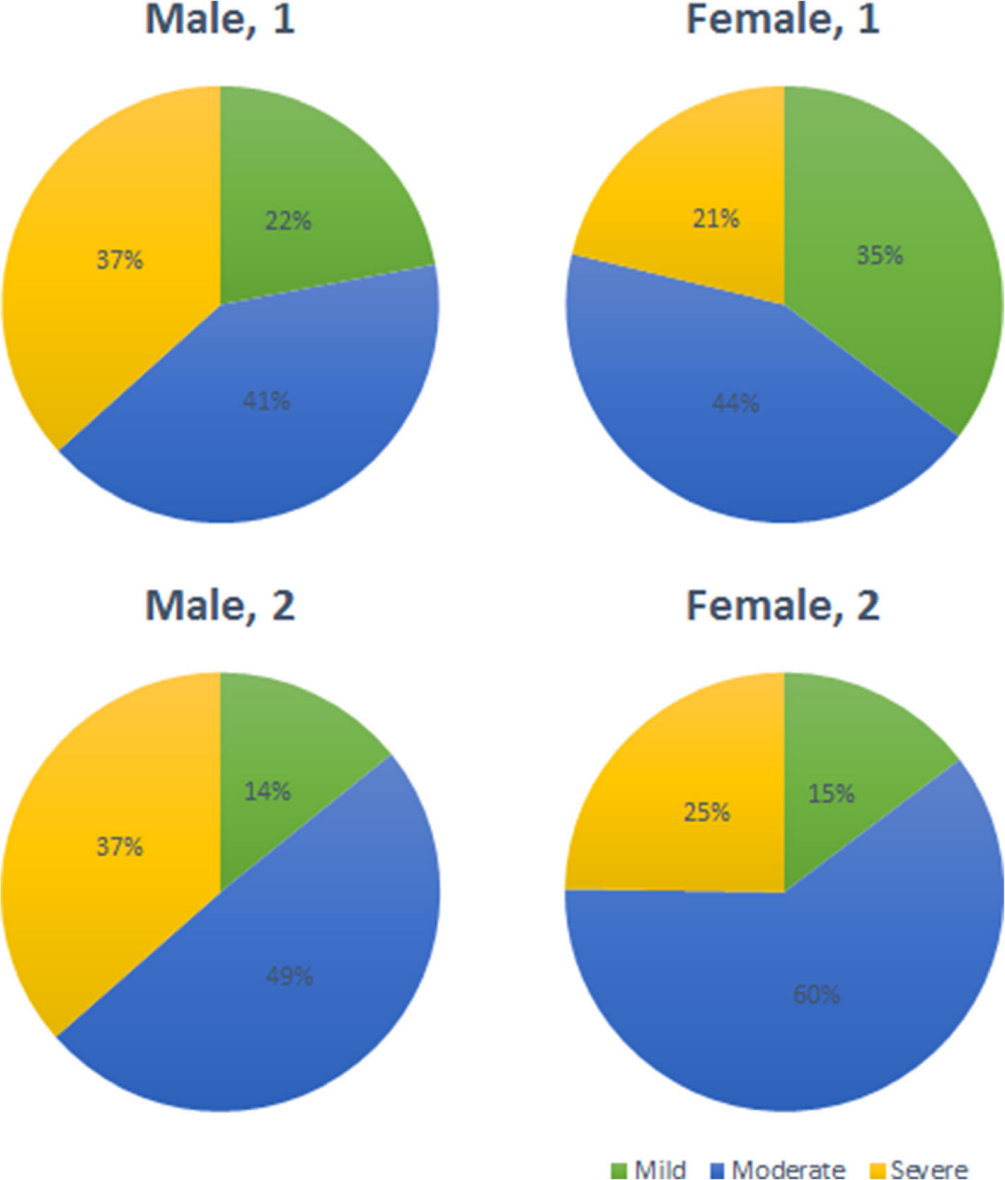
Severity distribution by sex of first and second series of COVID-19 inpatients. Legend. The pie charts on the top of the figure correspond to the first series and those at the bottom to the second series

**Fig. 3:**
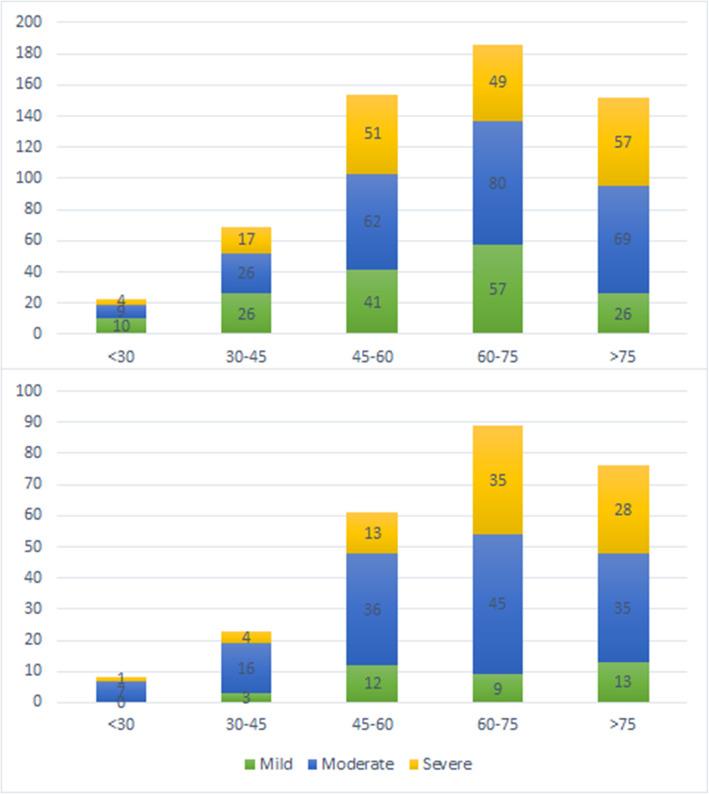
Severity distribution by age groups of first and second series of COVID-19 inpatients. Legend. The upper part of the figure corresponds to the first series and the lower part to the second series

Severity distribution within male inpatients was almost identical within the two series, but mild female cases decreased as moderate ones grew (*p* < 0.001, Fig. 2). Severity was significantly higher for all age groups during confinement, especially within the 60 to 75 years old group. Cases above 75 years were predominantly severe both at the beginning of the pandemic and during confinement. Mild inpatients were older in May (*p* = 0.01) than in March and so were (*p* = 0.028) severe ones (Fig. 3).

**Fig. 4:**
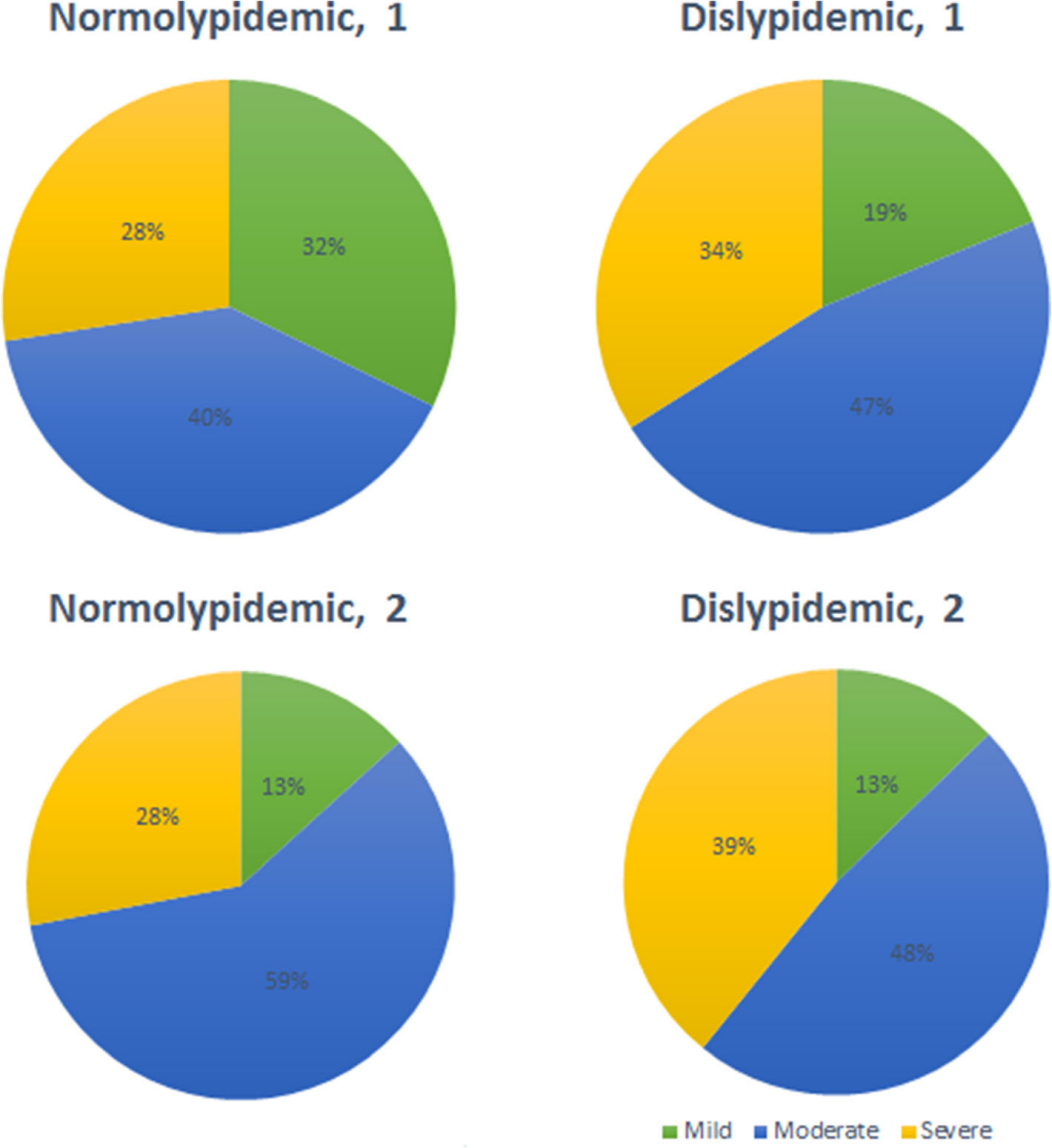
Severity distribution and dyslipidaemia of first and second series of COVID-19 inpatients. Legend. The pies on the top of the figure correspond to the first series and those at the bottom to the second series

Normolipidemic cases were less frequently mild to become moderate in May (*p* < 0.001) as compared to March (Fig. 4).

Regarding raw data stratified by severity, some laboratory parameters such as IL-6, CRP, ferritin and LDH, although high, showed values significantly lower than those in phase one. IL6, CRP and ferritin upon arrival were lower in May moderate cohort and CRP as well in mild inpatients. LDH was lower in May mild group as compared to that of March. In the same line, the lowering of CD8 cell count was more evident in phase two but did not reach the significance. All the other parameters compared yielded similar results in the first and second cohorts (data not shown).

#### Diagnostic accuracy of laboratory parameters

To look for cut-off points in laboratory parameters at admission that would allow us to predict the severity of COVID-19 disease, data from the two series of patients were pooled. Kurtosis and asymmetry were calculated for both cohorts and both fell apart > 10% for every parameter but age, lymphocyte count and LDH. In terms of diagnostic accuracy, only IL-6, CRP, LDH levels and lymphocyte CD4+ count offered a reliable area under the curve. The optimal cut-off and the diagnostic statistics for each parameter are shown in Table 5. Moreover, two extreme thresholds were determined in the merged cohort. A threshold with a likelihood ratio positive (LR+) appropriate for predicting severe COVID-19 and a point with a likelihood ratio negative (LR-) suitable for discarding severe COVID-19 was calculated. Extreme cut-offs for each parameter are shown in Table 5. When assessing diagnostic accuracy, CD19 count had an unacceptable AUC of 0.59 (95% CI: 0.47–0.71).

**Table 5:** Diagnostic validity of laboratory parameters

		AUC^**a**^	AUC 95% IC^**b**^	LR + ^**c**^	LR-^**d**^	YI^**e**^	Sensitivity (%)	Specificity (%)
**IL6**^**f**^ **(pg/mL)**								
Best cut-off	69.08	0.70	0.64–0.76			0.31	51.24	79.86
Best cut-off for LR+	175.1			5.2			28.10	94.60
Best cut-off for LR-	5.56				0.19		97.52	12.59
**CRP**^**g**^ **(mg/L)**								
Best cut-off	97.3	0.67	0.65–0.73			0.28	62.18	66.05
Best cut-off for LR+	291.85			5.16			14.29	97.23
Best cut-off for LR-	7				0.21		97.90	11.62
**Ferritin (ng/mL)**								
Best cut-off	632.5	0.67	0.62–0.73			0.29	72.66	56.79
Best cut-off for LR+	2688.5			5.51			12.23	87.77
Best cut-off for LR-	162				0.17		97.12	16.62
**LDH**^**h**^ **(U/L)**								
Best cut-off	328.5	0.71	0.66–0.75			0.36	65.58	70.62
Best cut-off for LR+	574			5.34			17.21	96.78
Best cut-off for LR-	177.5				0.3		88.37	30.99
**D-dimer (ng/mL)**								
Best cut-off	1068	0.62	0.57–0.66			0.21	45.28	76.12
Best cut-off for LR+	34,744			4.62			1.89	99.59
Best cut-off for LR-	None						na.	na.
Leucocyte count (cells*10^**3**^**/μL)**								
Best cut-off	7.835	0.62	0.58–0.67			0.22	50.57	71.90
Best cut-off for LR+	15.875			5.14			9.89	98.08
Best cut-off for LR-	None						na.	na.
Neutrophil count (cells*10^**3**^**/μL)**								
Best cut-off	6.26	0.66	0.62–0.70			0.27	51.33	75.44
Best cut-off for LR+	13.04			5.37			12.17	97.74
Best cut-off for LR-	None						na.	na.
Lymphocyte count (cells*10^**3**^**/μL)**								
Best cut-off	0.725	0.61	0.57–0.65			0.18	39.92	77.66
Best cut-off for LR+	0.365			5.34			10.27	98.08
Best cut-off for LR-	None						na.	na.
**CD3 + CD4+ count (cells/μL)**								
Best cut-off	535.5	0.70	0.61–0.80			0.41	90.32	50.51
Best cut-off for LR+	95.74			4.79			9.68	97.98
Best cut-off for LR-	660.6				0.16		93.55	39.39
**CD4 + CD8+ count (cells/μL)**								
Best cut-off	162.83	0.63	0.52–0.75			0.22	58.06	63.64
Best cut-off for LR+	None						na.	na.
Best cut-off for LR-	None						na.	na.
**CD19+ count (cells/μL)**								
Best cut-off	na.^i^	0.59	0.47–0.71			na.	na.	na.
Best cut-off for LR+	na.						na.	na.
Best cut-off for LR-	na.						na.	na.
**Natural Killer count (cells/μL)**								
Best cut-off	147.5	0.65	0.54–0.77			0.28	75.00	53.26
Best cut-off for LR+	43.29			5.11			16.67	96.74
Best cut-off for LR-	229.38				0.18		95.83	22.83

*Abbreviations*: *AUC*^a^ Area under the curve, *AUC 96% IC*^b^ 95% Confidence Intervale of the area under the curve, *LR*^c^ Likelyhood ratio positive, *LR-*^d^ Likelyhood ratio negative, *YI*^e^ Youden Index, *IL6*^f^ Interleukin 6, *CRP*^g^ C-reactive protein, *LDH*^h^ Lactate dehydrogenase, *na.*^i^ Non aplicable

## Discussion

This study raises the question of whether the decision values for both clinical and laboratory parameters associated with severe COVID-19 might change, depending on external or environmental factors such as lockdown. Even working with the same population, selection criteria, and observers, a certain degree of data heterogeneity arises and searching for prognostic risk factors or laboratory cut-off values becomes troublesome. Noise factors should be therefore discarded to simplify and improve triage algorithms.

Regarding severity of SARS-CoV-2 infection, different risk factors or comorbidities and laboratory parameters have been reported to date, but none of them is consistent across published studies [27]. Our group has previously described the relationship among demographic, clinical and laboratory parameters with COVID-19 severity in a retrospective study including 584 patients infected for SARS-CoV-2, just before the alarm state declaration and the close confinement in our country in March 2020 [16]. In the present work, with the same case selection and severity criteria, we describe the relationship between clinical risk factors and laboratory parameters at admission in a group of 257 patients admitted to Spanish hospitals during confinement in May 2020.

Concerning clinical risk factors found at the first moment (age, male sex, hypertension, diabetes, hyperlipidaemia, ARBs intake) only age and male sex remain relevant in this second moment. The epidemiological background, which was not recorded in the first phase, was a risk factor for severest COVID-19. Laboratory parameters such as leukocytes, neutrophils, IL-6, CRP, ferritin, D-dimer, and LDH increased with the severity conversely to the decrease in lymphocyte percentage.

However, as comparing both cohorts, severity, and the presence of comorbidities such as dyslipidaemia and secondary immunodeficiency (SID) was significantly higher in patients hospitalized during confinement while the use of ARBs was significantly lower.

Strikingly, IL-6, CRP and ferritin, whose elevations were associated with greater severity in both phases, were lower in this second phase than in the first one. There is no straightforward explanation for this finding. One of the major differences that could be a decisive factor is that cases included in the first series were infected just before strict containment measures were enacted in Europe. Meanwhile, cases included in the second series were admitted during close confinement. We may guess that during strict confinement, the epidemiological background would have been oscillating to institutional contagions. In the second phase, the epidemiological background was recorded, and it is worth noticing that 25% of the cases were institutionalized in nursing homes. A higher number of institutionalized cases would mainly impact data because of their older age and a higher rate of second-stage patients’ pre-existing morbidities. In turn, the relative weight of the age-associated factors might be even higher in this series. Therefore, the relative weight of other factors, such as those associated with the viral infection, might be lower. Additionally, medical prescribing habits for such as the use of RAASBs might have been influenced either by institutionalization or by the highly changing huge number of variable-quality scientific reports at the beginning of pandemics.

A remarkable effort has been made to identify clinical and laboratory factors that can help us predict the evolution of COVID-19 disease. This effort crystallized in a vast amount of original articles and meta-analyses. As in our previous study, in most meta-analyses, age, as well as the presence of hypertension, diabetes and cardiovascular disease, are identified as COVID-19 risk factors [17, 18]. Regarding laboratory data upon admission, in line with our findings, several studies reveal a significant increase in IL-6 [17, 27–29], CRP [17, 27–29], D-dimer [17, 18, 27, 28], ferritin [17, 29], LDH [17, 18], leukocytes [17, 28, 29], neutrophils [27–29], and a decrease of lymphocytes [17, 18, 27–30], a decrease of T lymphocytes CD4+ [27, 29, 30] and CD8+ [18, 27, 29, 30], related to the severity. However, in some of these papers [17, 28] the authors warn about heterogeneity across different studies selected for meta-analysis, pointing out as possible causes the origin of the data, the sample size and the month of publication.

In our current work, some previously identified factors associated with severity along the first period lost their significant relationships while age and gender were consolidated as severity factors. In addition to remark, age remains a determinant of the main comorbidities initially identified as risk factors. Furthermore, significant quantitative differences within laboratory values have been detected as comparing both periods, pointing out a temporal bias.

Particular attention has been paid as well to clarify the role of RAASBs in SARS-CoV-2 infection and the severity of COVID-19 disease. Several meta-analyses have addressed this central issue, but no consensus is met to date. An overall protective effect of RAASBs use is described, this would be mainly attributable to the use of ACEIs (OR:0.652; 95% CI: 0.478–0.891), but not similar effect is observed with concomitant ACEIs plus ARBs (OR:0.867; 95% IC:0.638–1.179) or ARBs alone intakes (OR:0.810; 95% IC:0.629–1.044) [31]. In another meta-analysis where the relationship of the use of RAASBs with the probability of COVID-19 is stated, geographical differences are evidenced, detecting that the use of RAASBs is generally associated with a better prognosis only in studies carried out in Asian countries (OR.0.37; 95% CI: 0.16–0.89) whereas, in those carried out in North America, it is commonly associated with an even more significant increase in ICU admissions (OR: 1.75; 95% CI: 1.37–2.23) and in those carried out in Europe it is related with a higher death probability (OR: 1.68; 95% CI: 1.05–2.70). The authors note that ACEIs would be mainly protective and conversely, ARBs would be associated with an increased risk of death [32]. In a different sense are the findings of the meta-analyses of Megaly et al. [33] and Chan et al. [34]. In the first one, the use of RAASBs is associated with a lower risk of death (OR: 0.57; 95% CI: 0.32–0.98) [33]. In the latter, the use of RAASBs is not globally associated with an increased risk of infection (ACEIs OR: 0.95; 95% CI: 0.86–1.05), (ARBs OR: 1.05; 95% CI: 0.97–1.14) [34]. However, ARBs increase the risk of infection in young subjects, while ACEIs do not increase the susceptibility to infection, not the severity or mortality from COVID-19 [34]. In the phase 1 of our study, a protective effect promoted by ACE inhibitors’ intake was described, while the use of ARBs was associated with increased severity [16]. However, our current study does not find any effect of the use of RAAS inhibitors, neither protection nor higher risk. Due to the widespread use of this drug and the apparent beneficial effects concerning COVID-19 disease of ACEIs compared with the deleterious effect of ARBs, more controlled studies are necessary to delve into this major concern.

In order to search for cut-off points of laboratory parameters at the time of admission, which would help us predict the evolution of the patients, data from both series were merged. In terms of diagnostic accuracy, only IL-6, LDH, CRP and CD4 + lymphocyte counts offered an acceptable area under the curve (Table 5).

Accordingly, the extreme threshold values that would allow us to confirm or rule out a serious evolution, for these parameters or the NK count were only informative. On the other hand, leukocyte, neutrophil and lymphocyte counts, although predictable, yielded cut-off points with adequate LR+, that could help foresee a severe evolution.

Several studies analyse the ability of cut-off points in laboratory parameters to predict the evolution of COVID-19. For LDH, several cut-off points have been described. LDH (250–500 U/L) (HR 2.5, 95% CI 1.2–5.2) and LDH > 500 U/L (HR 9.8, 95%CI 2.8–33.8) [20]; LDH > 277 U/L (sensitivity 58.7%, specificity 82% for severe disease) and 359.5 U/L (sensitivity 93.8%, specificity 88.2% for death) [35]; LDH > 450 U/L (AUC 0.76, sensitivity 75%, specificity 70%) for respiratory failure) [36]; LDH > 325 U/L (AUC 0.762 for severe disease) [23]. In our study the optimal cut-off for LDH was similar to those previously described, 328.5 U/L (AUC 0.71, 95% CI:0.67–0.75) (Sensitivity: 66.6%; Specificity: 75.2%) for severe disease (Table 5). Additionally, two extreme thresholds were calculated; the value 574 IU/L had an LR+ of 5.3 for diagnosing severe disease, and a value of 177.5 IU/L had an LR- of 0.30 for discarding severe COVID-19.

Several cut-off points have been described for the CRP. A value of 11 μg/dL showed an AUC of 0.78 (sensitivity 72%, specificity 71%) for respiratory failure [36]; CRP > 25.95 mg/L has an AUC of 0.84 (95% CI 0.780–0.905) for severity [37]; CRP > 46 mg/L has an AUC of 0.777 for severity [23]; CRP > 38.2 mg/L has and AUC of 0.875 (95% CI 0.867–0.883) (sensitivity 84.6%, Specificity 92.3%) for severity [28]. In our study the optimal CRP cut-off was higher than those previously published, 97.3 mg/L (AUC 0.69, 95% CI 0.65–0.73) (Sensitivity: 62.2%; Specificity: 66.1%) (Table 5). Concerning the extreme thresholds, the value 291.85 mg/L had an LR+ of 5.16 for diagnosing severe disease and a value of 7 mg/L had an LR- of 0.21 for discarding severe COVID-19.

Like other acute phase reactants, ferritin is elevated in the moderate and severe forms of COVID-19. Tahtasakal et al. have proposed a ferritin value > 303 μg/L (303 ng/mL) (AUC 0.698) as a predictor of severity [23]. Ferritin > 200 ng/mL is also part of a model to predict patients who will need high-flow O2 input (HR 7.5) [4]. In the present study, ferritin had an AUC of 0.67 (95% CI:0.62–0.72), and the best cut-off was much higher than those reported in the previous publications, 632.5 ng/mL (Sensitivity: 72.7%; Specificity: 56.8%) (Table 5). Concerning the extreme thresholds, the value 2688.5 ng/mL had an LR+ of 5.51 for diagnosing severe disease and a value of 162 ng/mL had an LR- of 0.17 for discarding severe COVID-19.

Zhou et al. have proposed a D-dimer value > 1 mg/L (1000 ng/mL) (OR 18.42, 95% CI 2.64–128.55) for COVID-19 associated mortality [19]. The optimal cut-off proposed by Tahtasakal et al. is 574 μg/L (574 ng/mL) for severe COVID-19 (AUC 0.694) [23]. Elshazli et al. in a meta-analysis have found 0.48 μg/L (480 ng/mL) as the optimal value for predicting severity (AUC 0.876, 95% CI 0.868–0.884) (sensitivity 88.9%, specificity 77.8%) [28]. A value of 0.65 mg/L (650 ng/mL) has been proposed by Zhang et al. as a predictor for severity in older adults [38]. In our study, D-dimer had an AUC of 0.62 (95% CI:0.57–0.66) and the optimal cut-off was 1068 ng/mL (Sensitivity: 45.3%; Specificity: 76.1%) (Table 5). The extreme high threshold showed no utility because of their obviousness, 34,744 ng/mL with an LR+ of 4.6 for diagnosing severe disease. No D-dimer value had a reliable LR- less than 0.5 for discarding severe COVID-19.

IL-6 levels are used in the context of COVID-19 disease for patient follow-up and clinical decision-making. Several levels of IL-6 have been proposed in different studies to predict severity progression. In the meta-analysis by Elshazli et al. a cut-off point of 22.9 pg/mL obtained an AUC of 0.63 (95% CI 0.616–0.648) (Sensitivity 71.4%, Specificity 71.4%) [28]. A similar cut-off point, 34.9 pg/mL, showed an AUC of 0.760 and an OR of 12.750 (95% CI 2.2–75.3) to predict ICU admission [3]. A 64 pg/mL cut-off point for IL-6 is also part of a model to predict patients who will need high-flow O2 input (hazard ratio 18) [4]. A higher cut-off, 163.4 pg/mL, has been proposed for predicting death with a 91.7% sensitivity and 57.6% specificity [39]. In our study a value of 69.08 pg/mL has been found as a predictor of severity (AUC 0.70, 95% CI:0.64–0.76) pg/mL (Sensitivity: 51.2%; Specificity: 79.8%) (Table 5). Concerning the extreme thresholds, the value 175 pg/mL had an LR+ of 5.2 for diagnosing severe disease and a value of 5.56 pg/mL had an LR- of 0.19 for discarding severe COVID-19.

An elevated neutrophil count, lymphopenia, and an elevated neutrophil/Lymphocyte ratio are characteristic of severe COVID-19 [23, 28, 40]. Lymphopenia is also part of the infection pathogenesis and is both a cause and a consequence of the severity [41]. Ji et al. have proposed a cut-off point of 1*10^3^ lymphocytes/μL for the diagnosis of severe disease (HR 3.7, 95% CI 1.8–7.8) [20]. Tahtasakal et al. have proposed a cut-off point of 1.04*10^3^ cells/μL for the diagnosis of severe disease (AUC 0.678) [23]. In a meta-analysis with 6320 patients, a cut-off point of 0.98*10^3^ cells/μL (AUC 0.867, 95% CI 0.861–0.873) is proposed (sensitivity 81.2%, specificity 87.5%), as a marker of severe COVID-19 [28]. In our study, the AUC of this parameter was low 0.61 (95% CI:0.57–0.65) and the best cut-off was 0.725 cells*10^3^/μL (Sensitivity: 39.9%; Specificity: 77.7%) (Table 5). Regarding extreme thresholds, the value 0.365 cells*10^3^/μL had an LR+ of 5.34 for diagnosing severe disease. No lymphocyte count value had a reliable LR- under 0.5 for discarding severe COVID-19. In summary, both the low AUC and the absence of extreme values with adequate LR- mean that no reliable lymphocyte count values were found to help predict evolution in our study.

Total lymphocyte count, lymphocyte populations, especially the T ones, are affected in the severest cases of COVID-19. Different cut-offs for CD8+ lymphocytes have been reported. Du et al. [42] propose a cut-off point of 75 cells/μL to predict a fatal outcome. In our study, the CD3 + CD8+ count had a low AUC of 0.63 (95% CI:0.52–0.75) and the best cut-off was 163 cells/μL (Sensitivity: 51.6%; Specificity: 75%) (Table 4). Concerning the extreme thresholds, no CD3 + CD8+ count had an acceptable LR+ for diagnosing severe disease nor LR- less than 0.5 for discarding severe COVID-19. For CD4+ T-lymphocytes, there are few studies with predictive cut-off points, Zhang et al. have proposed a cut-off point of 268 cells/μL (AUC 0.804, 95% CI 0.695–0.912) for predicting severe disease in older adults with COVID-19 [38]. However, in our study. CD3 + CD4+ count had an AUC of 0.70 (95% CI:0.61–0.80) and the optimal cut-off was 535 cells/μL (Sensitivity: 90.3%; Specificity: 50.5%) (Table 4). The value 95.74 cells/μL had an LR+ of 4.79 for diagnosing severe disease, and a value of 660.6 cells/μL had an LR- of 0.16 for discarding severe COVID-19.

Finally, age has been ratified as a crucial factor in COVID-19 severity in our series. Age correlates with endothelial damage and coagulation dysfunction, immunosenescence, inflammaging, including the effects of chronic cytomegalovirus infection, increased prevalence of COVID-19-associated comorbidities, and low levels of vitamin D [43]. Immunosenescence refers to age-related changes in the immune system [44]. Older individuals are more susceptible to infections due to immunological changes associated with the ageing process [45]. These progressive changes affect both innate and adaptive immunity. They include a decrease in naive lymphocytes, the contraction of lymphocyte repertoire, increased memory lymphocytes, fibrotic changes in lymph node architecture, and dysregulation in cytokine production [46]. The low-grade chronic inflammatory state that accompanies ageing, called inflammaging, may predispose older adults to severe COVID-19 by impairing the immune response to SARS-CoV-2. Inflammaging is characterized by high levels of acute-phase proteins and pro-inflammatory cytokines [47]. It has been suggested that individuals with more severe SARS-CoV-2 infection may have a cytokine storm syndrome characterized by increased levels of cytokines and chemokines [45]. Cytokine storm in elderly with severe SARS-CoV-19 is associated with age-related pathophysiological processes, including senescent cell inflammatory phenotype, excess oxygen radical production, immunosenescence, and lack of vitamin D [48]. Endothelial damage is a critical point that allows us to identify patients prone to develop severe COVID-19. Endothelial barrier mechanisms are independently compromised by diabetes, obesity, age [49] and hypertension, that are known to determine bad COVID-19 prognosis. The hypothesis that attributes the severity of COVID-19 evolution to age-related changes is partly speculative, but supported by different experimental studies. Thus, Rydyznski et al., studying the specific humoral and cellular response to SARS-Cov-2, point out that age correlates with a more severe specific antigen immune response. Older individuals present an uncoordinated humoral and cellular response to SARS-CoV-2. This coordination is notably affected in those over 65 years of age. T lymphocytes’ shortage is associated with age and worse COVID-19 prognosis [50].

Baas et al. performing genomic analysis of the response to SARS-CoV-1 in a murine model, point out that older individuals present an exacerbated immune response and that the expression of the genes of TNF-α, IL-6, CCL2, CCL3, CXCL10 and INF-γ exhibit a biphasic pattern that correlates with the peak of viral replication and with the flow of lymphocytes to the areas of more severe histopathological damage in the lungs [51]. Sims et al. characterize the cytokine storm that accompanies severe COVID-19 and find a panel of markers, such as IL-6, PTX3, IL-1RA, CTSL1, IL-18 and RAGE that would reflect vascular endothelial disruption [52]. An unbalanced production of pro-inflammatory cytokines has been described in immunosenescence in healthy individuals. Thus, Shurin et al. demonstrate that INF-γ-inducible chemokines (MIG and IP-10) increase with age [53]. Concerning the above, Tincati et al. analysing the phenotype of cytokines and chemokines that characterizes the worsening of COVID-19 in the second week of the disease, point out that this critical point in the evolution of the disease is associated to higher levels of CXCL8/IL-8, CXCL-9/MIG and CXCL10/IP-10, and that the presence of circulating neutrophils is associated to these levels [54]. Likewise, Xiong et al., employing the transcriptomic analysis of the characteristics of bronchoalveolar lavage and peripheral blood mononuclear cells of individuals with COVID-19 pneumonia, point out the association between the pathogenesis of the disease and the excessive release of cytokines such as CCL2/MCP-1, CXCL10/IP-10, CCL3/MIP-1A and CCL4/MIP-1B [55]. Moreover, IP-10 (CXCL10) [56, 57] and MCP-1 [56] have been proposed as biomarkers related to the risk of death in COVID-19 [56] and severity [57]. In the autopsies of the patients who died due to COVID-19, besides the mononuclear inflammatory infiltration, the diffuse alveolar damage and the formation of hyaline membranes, the particular presence of vascular affectation with epithelial damage points to a probable direct cytopathic role of the virus stands out [58]. A hypothesis could be built, where the changes associated with ageing, such as epithelial dysfunction and changes in basal levels of cytokines and chemokines (standing out CXCL-10/IP-10 and CCL2/MCP-1) would enhance an exaggerated response triggered by the direct cytopathic action of the virus on endothelium. The specific response against SARS-CoV-2, when uncoordinated due to ageing, would contribute to a worse evolution.

## Conclusions

Some reliable and informative parameter cut-offs could help clinical coping with COVID-19 but lockdown data can rise heterogeneity and should be therefore cautiously managed.

The relationship between the use of RAABs and COVID-19 severity although widely commented, is not conclusively established. Controversial results should guarantee future controlled studies.

Age plays a pivotal role in COVID-19 severity, but not other age-related comorbidities. The hypothesis can be drawn of ageing related changes (epithelial dysfunction and basal levels of cytokines and chemokines) enhancing an exacerbated response to the direct cytopathic action of SARS-CoV2 on vascular endothelium. A specific but uncoordinated immune response against SARS- CoV-2 would determine a bad COVID-19 prognosis.

## Methods

### Study design and participants

A retrospective multicentre analysis was performed on a consecutive set of SARS-CoV-2 infected inpatients, microbiologically confirmed by positive polymerase chain reaction (CRP) test, admitted to the 13 hospitals, during May 2020. Cases were tracked for a five-week follow-up period from admission to discharge. A minimum sample size of 20 patients was considered for every hospital. A total of 260 individuals over 18 years old, from 13 Spanish hospitals were recruited. After data quality assessment, 257 patients were included in the analyses. Cases were stratified into three severity groups, when recorded for inclusion in this study according to the following criteria, adapted from the technical document published by the World Health Organization [59].

- Mild: whenever clinical symptoms were mild with no abnormal radiological findings.
- Moderate: cases with confirmed pneumonia that was not considered severe
- Severe: when at least one of the following criteria was met: acute respiratory distress, shock, admission to the intensive care unit (ICU), the process was so considered by the physician in charge. Any death was as well classified as severe.

This retrospective observational study was conducted according to national regulations, institutional policies and in the tenets of the Helsinki Declaration. It was approved by the local institutional Ethics Committee of any involved hospitals.

Data from 584 case records from the previously analysed series [16], were compared with data from the 257 cases in the present study and both were pooled to explore cut-off values.

### Data collection

Any data analysed were extracted from electronic medical records. The collection form included demographic, epidemiological and clinical data: age, sex, history of diabetes mellitus (DM), dyslipidaemia, hypertension (HTA), renin-angiotensin-aldosterone system blockers (RAASBs) and statins intake, the smoking status, obesity, time from onset to diagnosis, laboratory data on admission, and COVID-19 severity using the criteria previously defined.

Additionally, data from a former study conducted by our group the pre-confinement phase [16] were compared to those in the current study and merged for cut-off analyses. Inclusion and severity criteria were the same for both cohorts.

### Laboratory data

Major laboratory markers were extracted from medical records on admission. Routine blood examinations included leukocyte, neutrophil and lymphocyte counts (cells/μL) and percentages. Serum biochemical tests recorded were ferritin (μg/L), lactate dehydrogenase (LDH, U/L), C- reactive protein (CRP) and D-dimer (μg/L). Immunological tests recorded were interleukin-6 (IL6, pg/mL), Lymphocyte population count (cells/μL) and percentage, complement factors C3 and C4 and immunoglobulins IgG, IgA and IgM (mg/dL).

### Statistical analysis

Demographic and clinical characteristics of patients were expressed as their mean and standard deviation (SD); when not adjusting to a normal distribution, the median was used to represent non-parametric data for continuous variables and frequency distributions represented categorical variables.

Kolmogorov-Smirnov test was performed on each continuous variable to contrast normality. To analyse the overall differences between the three groups: mild, moderate, and severe type, the ANOVA was tested on variables with normal distribution and *n* > 30 (% and CD4 lymphocyte count, % of CD8 lymphocytes, % of NK and C3 concentration). The Kruskal-Wallis test was used to analyse the relationship with severity for non-parametric variables. To contrast the “Ho” of independence within categorical variables, Chi-square and Fisher’s exact test were used.

A comparison of data collected during the lockdown period with our published data collected outside the lockdown [16] was performed as independent samples’ Wilcoxon test for medians.

Pooling data from the two series searching for predictive cut-off values: We have previously published the association of laboratory parameters with severity from a cohort of COVID-19 hospitalized patients before complete lockdown. We merged the data from this cohort with those obtained during our country home confinement. To determine the diagnostic validity of each laboratory parameter, sensitivity, specificity, the receiver operating curve (ROC), the area under the curve (AUC) and the optimal cut-off (Youden index) were calculated. The maximum value of the Youden index can be used as a criterion for selecting the optimal cut-off value when a diagnostic test is expressed as a numerical value [60]. Moreover, to maximize the specificity (to rule in severe COVID-19) and the sensitivity (to rule out severe COVID-19), likelihood ratio positive and negative were calculated. A diagnostic test will be more beneficial to the extent that its positive likelihood ratio (+LR) is of greater magnitude since it allows confirming with certainty the presence of disease, and its -LR has a low value since it rules out the disease [61].

## Supplementary Information

**Additional file 1: Supplementary Table 1.** Comparison between Phases I and II for clinical and demographic characteristics.

**Additional file 2: Supplementary Table 2.** Comparison between Phases I and II for Age and Laboratory results.

## Data Availability

All collected data, including fully anonymized participant data, are available to others. Available information includes fully anonymized participant data and data dictionary. Related documents are available from the date of publications henceforth: study protocol, statistical analysis, and approval of the ethical board. These documents are available from the date of publications henceforth at email address cmartinalo@saludcastillayleon.es or aurora.jurado.sspa@juntadeandalucia.es Data will be shared after approval of proposals by the Valladolid Este Ethical Committee.

## Abbreviations

ACEIs: Angiotensin-converting enzyme inhibitors
ARBs: Angiotensin II receptor blockers
RAASB: Renin-angiotensin-aldosterone system blockers
LDH: Lactate dehydrogenase
CRP: C- reactive protein
IL-6: Interleukin-6
NK: Natural Killers
IgG: Immunoglobulin G
IgA: Immunoglobulin A
IgM: Immunoglobulin M
PCR: Polymerase chain reaction
SD: Standard deviation
IQR: Interquartile range
ROC: Receiver operating curve
AUC: Area under the curve
LR: Likelihood ratio
